# Spatial prediction of *Plasmodium falciparum *prevalence in Somalia

**DOI:** 10.1186/1475-2875-7-159

**Published:** 2008-08-21

**Authors:** Abdisalan M Noor, Archie CA Clements, Peter W Gething, Grainne Moloney, Mohammed Borle, Tanya Shewchuk, Simon I Hay, Robert W Snow

**Affiliations:** 1 Malaria Public Health & Epidemiology Group, Centre for Geographic Medicine Research-Coast, Kenya Medical Research Institute/Wellcome Trust Research Programme, P.O. Box 43640, 00100 GPO, Nairobi, Kenya; 2 Centre for Tropical Medicine, University of Oxford, John Radcliffe Hospital, Headington, Oxford, OX3 9DU, UK; 3 School of Population Health, University of Queensland, Brisbane, Queensland, 4006, Australia; 4 Centre for Geographic Health Research, School of Geography, University of Southampton, Southampton, SO17 1BJ, UK; 5 United Nations Food and Agricultural Organization, Food Security Analysis Unit-Somalia, 3rd Floor, Kalson Towers, Parklands, P.O. Box 1230, Village Market, Nairobi, Kenya; 6 United Nations Children's Fund, Somalia Support Centre, P.O. Box 44145, 00100, Nairobi, Kenya; 7 Spatial Ecology and Epidemiology Group, Tinbergen building, Department of Zoology, University of Oxford, South Parks Road, Oxford, UK

## Abstract

**Background:**

Maps of malaria distribution are vital for optimal allocation of resources for anti-malarial activities. There is a lack of reliable contemporary malaria maps in endemic countries in sub-Saharan Africa. This problem is particularly acute in low malaria transmission countries such as those located in the horn of Africa.

**Methods:**

Data from a national malaria cluster sample survey in 2005 and routine cluster surveys in 2007 were assembled for Somalia. Rapid diagnostic tests were used to examine the presence of *Plasmodium falciparum *parasites in finger-prick blood samples obtained from individuals across all age-groups. Bayesian geostatistical models, with environmental and survey covariates, were used to predict continuous maps of malaria prevalence across Somalia and to define the uncertainty associated with the predictions.

**Results:**

For analyses the country was divided into north and south. In the north, the month of survey, distance to water, precipitation and temperature had no significant association with *P. falciparum *prevalence when spatial correlation was taken into account. In contrast, all the covariates, except distance to water, were significantly associated with parasite prevalence in the south. The inclusion of covariates improved model fit for the south but not for the north. Model precision was highest in the south. The majority of the country had a predicted prevalence of < 5%; areas with ≥ 5% prevalence were predominantly in the south.

**Conclusion:**

The maps showed that malaria transmission in Somalia varied from hypo- to meso-endemic. However, even after including the selected covariates in the model, there still remained a considerable amount of unexplained spatial variation in parasite prevalence, indicating effects of other factors not captured in the study. Nonetheless the maps presented here provide the best contemporary information on malaria prevalence in Somalia.

## Background

Maps of disease distribution are an essential tool for optimizing the allocation of resources for malaria interventions [1,2]. There have been a number of attempts to develop malaria transmission maps at different geographic scales based on expert opinion [3,4]; deterministic biological models driven by the conceptual relationship between transmission and environmental covariates [5]; and empirical transmission models based on entomological inoculation rates [6,7] or human infection prevalence data [8-17]. These methods suffer several limitations: expert opinion maps are subjective; deterministic models ignore the secular effects of expanded coverage of interventions that supersede the influence of climate on the epidemiology of malaria and do not quantify uncertainty around model results. Where studies have used observational data to predict malaria distributions, most have used historical data collected opportunistically from secondary sources [10,15,16] that did not involve random sampling and/or a sampling framework optimized for spatial analysis.

Arguably the greatest need for malaria maps is at the periphery of stable, endemic areas where decisions about the delivery of standard suites of interventions, such as those promoted by the Roll Back Malaria (RBM) initiative to support malaria control in high transmission areas, may become less appropriate or cost-efficient. In areas of perceived low malaria risk there is little empirical information on the risks and intensity of transmission. As such the semi-arid regions of the horn of Africa remain less well described epidemiologically compared to the rest of malaria endemic sub-Saharan Africa (SSA) and there are no contemporary national maps of the extents of malaria risk. The Malaria Atlas Project (MAP) while maintaining a global remit in its efforts to improve the cartography of malaria [2] is equally committed to developing national mapping initiatives with country partners, where the data available can support rigorous cartography. Somalia represents the first such example.

A *Plasmodium falciparum *malaria prevalence map for Somalia is presented here using Bayesian geostatistical analysis of community-based parasite prevalence survey data. The data used in this analysis have several unique features that minimize some of the problems of using retrospectively assembled data: first the community data were derived from random sample surveys undertaken as part of national malaria or nutritional surveys; second all the data were collected using similar methodologies; and finally all the data represent contemporary infection prevalence between 2005 and 2007.

## Methods

### Country context

**Figure 1:**
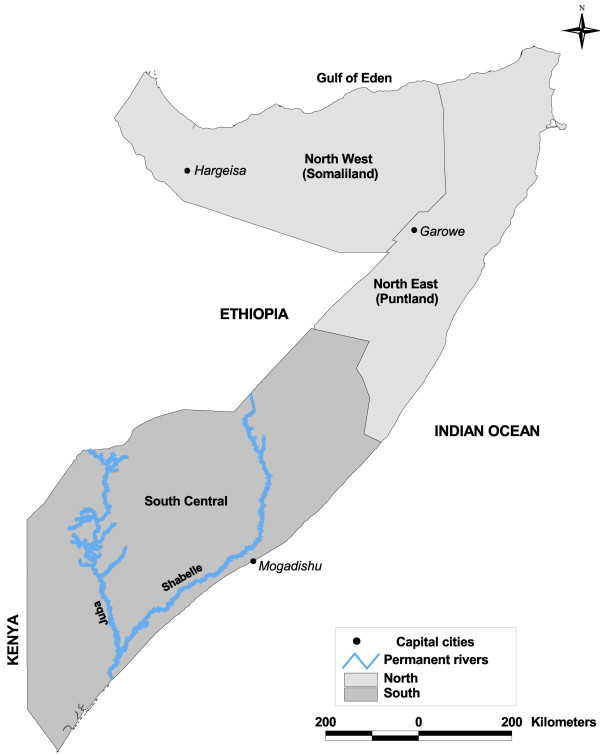
Map of Somalia showing the self-declared states of Puntland, Somaliland and South Central; their capital cities; and the two main rivers of Juba and Shabelle.

Somalia is located in the horn of Africa with a predominantly semi-arid climate and is transected by two major seasonal rivers, the Shabelle and the Juba (Figure 1). Somalia is divided into three zones: South-Central; North-West (Somaliland); and North-East (Puntland) (Figure 1). The country has been without a functioning national government for almost two decades. Several international relief agencies and non-governmental organizations, however, support the provision of public services including health services [18]. While not internationally recognized, the three zones are all self-declared states, each with independent "ministries of health". In collaboration with these ministries of health, the United Nations Children Fund (UNICEF), as the principal recipient of money from the Global Fund to Fight Aids TB and Malaria (GFATM), together with World Health Organization (WHO), are responsible for supporting malaria control activities. These activities include: training health workers and equipping health facilities; supply of anti-malarials; distribution of insecticide-treated nets; and funding of entomological and parasitological surveillance [19].

The earliest malariometric surveys undertaken in Somalia were in the North-West in 1946 which reported a highly varying prevalence distribution of *P. falciparum *ranging from 0 to 17% across three clusters of villages [20]. Between the 1940s and 2005 there were only three malaria infection surveys across five villages in the Lower Shabelle area of the south-central zone [21-23]. Based on limited entomological data, malaria transmission is thought to be supported almost entirely by *Anopheles arabiensis *[24-26].

### Assembling the survey data on parasite prevalence

Following the dearth of *P. falciparum *parasite rate (*Pf*PR) surveys over the last 50 years, community-based surveys of *Pf*PR have become a routine undertaking across the country since 2005. These surveys have been embedded in two major activities. First, a national malaria indicator survey was conducted by the WHO between January and February 2005 in the south-central and north-west zones [27] and in July 2005 in the north-east [28]. A stratified multi-stage random sampling strategy was adopted. Within each zone all regions were sampled and out of 120 districts in these regions, 88 were selected at random. Randomly selected villages within each district were surveyed successively until the required number of respondents of all ages (at least 845 per region) was achieved. Second, the United Nations Food Agricultural Organization-Food Security Analysis Unit (FAO/FSAU) completed 18 independent cluster sample surveys between March and November 2007. Malaria parasitology in all age-groups was included in these routine nutritional surveys at the request of UNICEF. In each survey a stratified multi-stage cluster sampling design was adopted where the sampling frame of a selected district was based on three livelihood definitions (pastoral, agro-pastoral, and riverine) [29,30] within which 30 rural communities and 30 households within each community were selected at random.

In all surveys, evidence of parasitaemia was determined using *P. falciparum *specific Rapid Diagnostic Tests (RDTs). WHO used ParaHIT – f™ Device (Span Diagnostics Limited, Surat, India) while FSAU used Paracheck Pf™ (Orchid Biomedical Systems, Goa, India). The purpose of the survey was explained to each household head or adult representative from whom informed consent was then sought prior to undertaking parasitological tests. All individuals who tested positive for infection were treated with nationally recommended first-line therapy [31]. An inclusion criterion of a minimum of 40 individuals per community was used to select villages to include in the analysis to minimize random variation inherent in small samples [32,33].

Survey data from all three sources were combined into a single database. Where communities had been surveyed more than once, the survey with the largest sample size was selected. A detailed search was undertaken to establish a set of spatial coordinates for each community. For some of the later surveys undertaken by FSAU, global positioning systems (GPS) were used to provide a longitude and latitude. For the remaining settlements a combination of electronic gazetteers [34,35] and other nationally derived UN sources of longitude and latitude [36] were used to locate the community. Finally, the location of each settlement was verified by using Google Earth (Google, Seattle, USA) to visually inspect whether the coordinates matched evidence of human settlement. Those settlements for which no reliable source of the coordinates could be obtained were excluded from the analysis.

**Figure 2:**
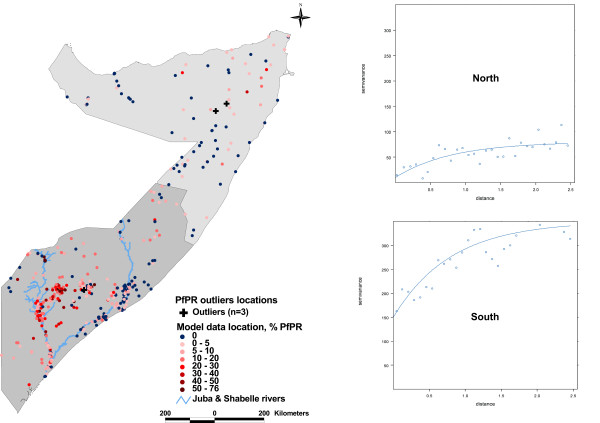
**Map and variograms of north and south of Somalia showing the distribution PR data locations (the X-axis shows distance as degrees latitude and longitude).** The data were distributed as model (n = 113 north; n = 336 south) and outlier (n = 2 north; n = 1 south) locations. Variograms are of the data after outliers were removed.

The assembled *Pf*PR data locations were not distributed evenly across Somalia with a higher concentration in the South-Central zone. Exploratory analysis showed different spatial autocorrelation structures for the two zones (Figure 2) and a single geostatistical model for the whole country was deemed inappropriate. To allow representative models to be developed in each region, the data were split and analysis was carried out separately for the north (north-west and north-east states combined) and the south (south-central state) (Figure 2).

### Outlier detection

Geostatistical methods are particularly sensitive to outlying values that exert a significant effect on predictions. Extreme outliers were therefore identified and excluded using a spatial filter. The method assumes that the probability of an unusually large *Pf*PR value being a genuine 'outlier' is larger if (a) it is in a neighbourhood of generally much smaller values and/or (b) the neighbourhood is generally uniform. A spatial filtering algorithm was implemented that incorporated these heuristic considerations (Additional File 1).

### Selection of covariates

Climatic and survey covariates were considered for inclusion in the spatial prediction model. The following four climatic variables were considered, each of which was resampled to 5 × 5 km resolution to be consistent with the prediction grid. 1) The *enhanced vegetation index *(EVI) derived from the global Moderate Resolution Imaging Spectroradiometer (MODIS) satellite imagery for the period 2001–2005 [37]. Temporal Fourier analysis was undertaken to derive a global EVI index [38]. These data were available for each month at 1 × 1 km spatial resolution and obtained from a global archive developed recently by the Spatial Ecology and Epidemiology Group of the Department of Zoology, University of Oxford [38]. Scaled EVI values ranged from 0–1, representing no, to complete vegetation cover. 2) *Precipitation and temperature *data described as the average monthly precipitation and temperature (minimum and maximum) at 1 × 1 km spatial resolution were downloaded from the WorldClim website [39]. These climate surfaces were developed through interpolation of global meteorological data collected from 1950–2000 [40]. 3) *Distance to permanent water bodies *was derived for each survey location using information provided by Africover [41] and those of marshes, flood plains and intermittent wetland from the Global Lakes and Wetlands databases [42]. 4) The effect of month of survey was assessed because of the observed temporal heterogeneity of *Pf*PR data. February was selected as the "reference month" for both zones as this was the earliest calendar month in which surveys were undertaken.

The annual mean of each environmental covariate (derived from the monthly data) was extracted at each survey location using ArcGIS 9.1 (ESRI Inc., USA). To assess the effects of the covariates on observed PfPR, non-spatial binomial logistic regression models were implemented in Stata/SE Version 10 (Stata Corporation, College Station, TX, USA). With PfPR as the dependent variable, bivariate binomial logistic regression models were fitted and covariates with Wald's P > 0.2 were excluded from subsequent analyses. Collinearity among all remaining covariates was assessed and if a pair had a correlation coefficient > 0.9, the variable with the highest value of Akaike Information Criterion (AIC) was discarded. To select which of the two temperature variables (maximum and minimum) to include into the multivariate model, the one with lowest value of AIC was chosen [9]. A non-spatial binomial multivariate logistic regression was then fitted, starting with a saturated model, and then seeking a parsimonious model using backwards variable elimination with an exit criterion of Wald's *P *> 0.2. Variables that exhibited non-linear relationships with PfPR were dichotomized at the median.

### Bayesian geostatistical models

Bayesian geostatistical (kriging) techniques provide a framework for predicting (interpolating) values of a variable of interest at unobserved locations given a set of spatially distributed data, incorporating spatial autocorrelation and computing uncertainty measures around model predictions [43,44]. Spatial autocorrelation in the Somalia *Pf*PR data was therefore first evaluated by computing empirical variograms, a graphical summary of spatial autocorrelation structure, separately for the south and the north. Different variogram structures were observed for the two zones indicating that a single stationary model for the whole country was inappropriate. Comparison of the variograms suggested greater heterogeneity of observed parasite prevalence data in the south than in the north consistent with expert opinion of the transmission dynamics across the country [26]. Consequently models were constructed separately for each zone. Bayesian binomial generalized linear geostatistical models [43] were implemented in each zone with the spatial component modelled as a stationary Gaussian process with mean of zero and covariance structure defined by a powered exponential function [45]. Because survey data were modelled as a conditionally binomial variable, given the underlying Gaussian process, the variance due to sample size was accounted for implicitly. The models were implemented in WinBUGS Version 1.4 (MRC Biostatistics Unit, Cambridge, UK). The models were constructed with and without the covariates in order to compare differences in model fit. Model fit was based on the deviance information criterion (DIC). Models with a smaller DIC value (with a difference >5) were considered to represent a better compromise between parsimony and fit [46]. The rate of decay of correlation between points (ϕ) with distance and the variance of the spatial process (σ^2^) were also recorded. The form of these models is shown in Additional File 2.

Predictions at non-sampled locations (defined over a regular 5 × 5 km grid overlaying the entire country) were made using the *spatial.unipred *function in WinBUGS which solves the model equation at each prediction location given the values of each covariate at the prediction location and the distance between prediction locations and observed data locations. Coefficients and model diagnostics were estimated using Markov Chain Monte Carlo (MCMC) simulations. The posterior probability distributions were used to classify prediction points to an endemicity class. Probability of class membership was computed as an additional measure to identify areas of high model uncertainties. For presentation purposes prediction maps from the best-fit model were combined for both zones. Continuous and categorical representations of predicted prevalence and probability maps were produced. The categorical classes of *Pf*PR selected were 0–<5% (low risk); 5–39% (medium risk); and ≥40% (high risk) and are based on a review of endemicity classification that would be most suitable as a guides for the likely impact of existing interventions [47].

### Model validation

A spatially de-clustered random sampling strategy was implemented to generate validation sets that could be considered spatially representative of the prediction space. Thiessen polygons, which enclose the area closest to a given point, were defined around each survey location. A 10% sample or a minimum of 30 survey locations (whichever was larger) were then drawn randomly for the north and the south with each data point having a probability of selection proportional to the area of its Thiessen Polygon. This meant data located in densely surveyed regions had a lower probability of selection than those in sparsely surveyed regions [48]. The Bayesian geostatistical models were then repeated without the validation dataset. Predicted *Pf*PR values from the Bayesian geostatistical models were compared to actual *Pf*PR values observed at the validation locations using the mean error (ME), mean absolute error (MAE) and the area under the curve (AUC) of the receiver operating characteristic (ROC). ME is a measure of the bias of predictions (the overall tendency to over or under predict) whilst MAE is a measure of overall precision (the average magnitude of error in individual predictions) and AUC is a measure of discriminatory ability of predictions with respect to a true prevalence threshold (observed endemicity classes). AUC values greater than 0.9 indicate excellent discrimination; >0.7 moderate discrimination; >0.5 poor discrimination; and <0.5, the model does not discriminate any more successfully than random allocation of test status [49,50].

### Ethical approval

Ethical approval was provided through permission by the Ministry of Health Somalia, Transitional Federal Government of Somalia Republic, Ref: MOH/WC/XA/146./07, dated 02/02/07. Informed verbal consent was sought from all participating households and individuals.

## Results

### Sample description

A total of 500 independent community data locations with sample size ≥ 40 were assembled from the two survey sources (Table 1). Of these, 48 (9.6%) communities with sample size ≥ 40 could not be spatially positioned and were excluded from the analysis. Of the remaining 452 survey locations, 115 (25%) were located in the north and 337 (75%) in the south (Table 1 and Figure 1). Two locations in the north and one in the south were detected as outliers (Table 1 and Figure 2). The mean *Pf*PR of the data, excluding the outliers, was 2.8% (95% CI: 2.1–4.6, n = 10,468) for the north and 11.4% (95% CI: 10.4–14.2, n = 20,011) for the south.

**Table 1:** Summary of Somalia survey data by source for the North and South zones.

	**Survey source**		
**Zone**	**FAO/FSAU** ** n (%)**	**WHO** ** n (%)**	**Total** ** n (%)**
**North**			
Number survey locations sampling with 40+ people	64	61	125
Number geo-referenced	55 (85.9%)	60 (98.4%)	115 (92.0%)
Number identified as outliers	0 (0.0%)	2 (3.3%)	2 (1.6%)
Number selected for model*	55 (85.9%)	58 (95.1%)	113 (90.4%)
Number of surveys with zero *Pf*PR**	32 (50.0%)	26 (42.6%)	58 (46.4%)
Population sample size	5,213	5,255	10,468
Number *Pf*PR positive	97	196	293
Mean (Median) *Pf*PR (%)	1.8 (0.0)	3.7 (1.1)	2.8 (0.0)
IQR *Pf*PR (%)	(0.0, 3.1)	(0.0, 4.0)	(0.0, 4.0)
**South**			
Number survey locations sampling 40+ people	311	64	375
Number geo-referenced	279 (89.7%)	58 (90.6%)	337 (89.9%)
Number identified as outliers	1 (0.3%)	0 (0.0%)	1 (0.3%)
Number selected for model*	278 (89.4%)	58 (90.6%)	336 (89.6%)
Number of surveys with zero *Pf*PR**	73 (23.5%)	23 (35.9%)	96 (25.6%)
Population sample size	16,048	3,963	20,011
Number *Pf*PR positive	2,081	208	2,289
Mean (Median) *Pf*PR (%)	13.0 (4.0)	5.2 (2.0)	11.4 (3.0)
IQR *Pf*PR (%)	(0.0,10.0)	(0.0, 6.0)	(0.0, 9.0)

*Sample size, number positive and the mean, median and IQR of *Pf*PR include those surveys reporting zero number positive for *Pf*PR.

**At the margins of stable malaria transmission, zero *P. falciparum *parasites in a sample is not necessarily an indication of true absence of parasites but could also be due to low sample sizes or test equipment with low sensitivity [33]. Given this difficulty in distinguishing true zero from sample zero at low *Pf*PR, surveys reporting zero prevalence were included in the analysis.

IQR = interquartile range; *Pf*PR = *Plasmodium falciparum *parasite rate; FAO/FSAU = Food and Agricultural Organization – Food Security Analysis Unit; WHO = World Health Organization.

### Non-spatial bivariate and multivariate analysis of covariates

Both minimum and maximum temperatures exhibited non-linear relationships with *Pf*PR and were dichotomized at the median. No pairs of covariates demonstrated collinearity. During the non-spatial bivariate logistic regression analysis EVI, precipitation, maximum and minimum temperature, distance to water and survey month all displayed a highly significant association with *Pf*PR and met the initial entry criteria for the non-spatial multivariate logistic models for both the north and south (Table 2). Minimum rather than maximum temperature was selected for inclusion. When the selected variables were examined together in the multivariate model, EVI was not significantly associated with *Pf*PR in the north (p = 0.223) or the south (p = 0.395) (Table 2). Excluding EVI and maximum temperature, all remaining variables were entered into the multivariate Bayesian geostatistical model (Table 3).

**Table 2:** Non-spatial bivariate and multivariate analysis of the association of survey and environmental covariates with *Pf*PR in north and south of Somalia.

	**Bivariate model**		**Multivariate model**	
**Zone**	**Odds ratio (95% ** **Confidence** ** Interval)**	**P-value**	**Odds ratio (95%** **Confidence** ** Interval)**	**P-value**

**North**				
Average annual Enhanced Vegetation Index	1.03 (1.06–1.11)	<0.001	1.97 (0.24–1.63)	0.223
Average annual precipitation	0.98 (0.97–0.99)	0.002	1.04 (1.02–1.08)	0.002
Average annual minimum temperature				
<median of 20.4°C	Ref	-	Ref	-
>median of 20.4°C	2.02 (1.59–2.58)	<0.001	1.35 (1.01–1.80)	0.045
Average annual maximum temperature				
<median of 32.4°C	Ref	-	Ref	-
>median of 32.4°C	2.63 (2.04–3.39)	<0.001	-	-
Distance to water features (km)	0.61 (0.49–.76)	<0.001	0.68 (0.46–0.99)	0.049
Survey month				
February	Ref	-	Ref	-
July	2.83 (2.24–3.59)	<0.001	3.24 (2.37–4.44)	<0.001
September	0.08 (0.04–0.16)	<0.001	0.10 (0.04–0.19)	<0.001
November	1.66 (1.29–2.14)	<0.001	1.58 (1.13–2.22)	0.008
**South**				
Average annual Enhanced Vegetation Index	1.09 (1.05–1.19)	<0.001	1.60 (0.54–4.70)	0.215
Average annual precipitation	1.03 (1.02–1.04)	<0.001	1.02 (1.03–1.04)	<0.001
Average annual minimum temperature				
<median of 22.1°C	Ref	-	Ref	
>median of 22.1°C	0.43 (0.39–0.48)	<0.001	0.61 (0.55–0.68)	<0.001
Average annual maximum temperature				
<median of 33.6°C	Ref	-	-	-
>median of 33.6°C	2.15 (1.96–2.36)	<0.001	-	-
Distance to water features (km)	1.04 (1.01–1.07)	0.005	0.84 (0.81–0.87)	<0.001
Survey month				
February	Ref	-	-	-
March	3.57 (3.25–3.92)	<0.001	7.62 (6.30–9.22)	<0.001
May	1.11 (0.95–1.30)	0.194	6.03 (4.68,7.79)	<0.001
June	0.73 (0.65–0.82)	<0.001	1.71 (1.43–2.04)	0.001
November	0.18 (0.14–0.24)	<0.001	0.33 (0.25–0.45)	<0.001
December	1.10 (0.99–1.22)	0.056	1.62 (1.41–1.87)	<0.001

Ref = reference or base outcome

**Table 3:** Summary output of Bayesian geostatistical models for the north and south of Somalia.

**Model/Variables**	**North**	**South**
**Bayesian geostatistical model (no covariates)**		
*α *(Intercept)	-4.62 (-5.44, -4.33)	-2.9 (-3.37, -2.27)
*ϕ *(Decay of spatial correlation (degrees latitude and longitude))	8.90 (3.11, 12.75)	4.79 (2.11, 6.97)
*σ*^2 ^(Variance of spatial process)	4.35 (271, 7.14)	7.14 (5.00, 8.76)
DIC	326	1,454
ME (% *Pf*PR)	3.83	4.14
MAE (% *Pf*PR)	4.12	5.06
AU-ROC*		
<5% *Pf*PR	0.72 (0.64, 0.86)	0.87 (0.72, 0.91)
5–39% *Pf*PR	0.66 (0.51, 0.80)	0.78 (0.66, 0.85)
≥ 40% *Pf*PR	NA	0.56 (0.37, 0.73)
**Bayesian geostatistical model (with covariates)**		
*α *(Intercept)	-4.62 (-5.23, -4.10)	-2.86 (-3.79, 2.27)
*ϕ *(Decay of spatial correlation)	10.35 (4.70, 12.88)	5.78 (2.95, 6.99)
*σ*^2 ^(Variance of spatial process)	3.70 (2.17, 7.14)	5.00 (3.70, 6.70)
DIC	323	1,429
ME (% *Pf*PR)	2.56	3.65
MAE (% *Pf*PR)	4.75	5.00
AU-ROC*		
<5% *Pf*PR	0.75 (0.64, 0.91)	0.91 (0.87, 0.99)
5–39% *Pf*PR	0.64 (0.43, 0.84)	0.81 (0.70, 0.94)
≥ 40% *Pf*PR	NA	0.51 (0.32, 0.83)
	**Odds ratio, (95% Bayes credible interval)**	
Month of survey		
Feb	**Ref**	**Ref**
Mar	-	4.06 (2.20, 7.63)
Jun	-	
Jul	3.25 (0.91, 11.36)	0.87 (0.48,1.46)
Sep	0.2 (0.02, 1.74)	
Nov	1.31 (0.33, 4.36)	0.48 (0.23, 0.96)
Dec	-	1.95 (0.91, 3.90)
Annual average minimum temperature		
<median of 20.4/22.1 (North/South)°C	**Ref**	**-**
>median of 20.4/22.1(North/South)°C	1.12 (0.84, 1.33)	0.83 (0.67,0.96)
Annual average precipitation	1.70(0.53, 5.44)	1.41 (1.07, 1.94)
Distance to water features (km)	1.22 (0.53, 2.81)	0.79 (0.74, 1.29)

DIC = deviance information criterion (measure of model fit); ME = mean error (measure of overall bias); MAE = mean absolute error (measure of overall precision); AU-ROC = areas under receiver operating characteristic. Values in parentheses are 95% Bayes credible intervals.

*There were only two survey locations in the validation set that had *Pf*PR ≥ 40% in the north region meaning reliable AU-ROC values could not be computed for this model.

### Bayesian geostatistical models

#### North

The Bayesian geostatistical model without covariates in the north had *σ*^2 ^(variance of spatial process) with a posterior median of 4.35 (95% credible interval (CI): 2.71, 7.14); *ϕ *(rate of decay of spatial correlation) of 8.90 (95% CI: 3.11, 12.75); a DIC value (measure of model fit) of 326; a ME (measure of model bias) of 3.83% *Pf*PR; and a MAE (measure of model precision) of 4.12% *Pf*PR (Table 3). The results of the multivariate Bayesian geostatistical model showed that none of the selected covariates remained significant (odds 95% CI included 1) after accounting for spatial correlation (Table 3). The DIC of the multivariate model was 323 and only marginally lower than that of the model without covariates, implying that the inclusion of the covariates did not improve overall model fit in the north. However, the covariates did account for some of the spatial variation in the data with spatial variance (*σ*^2^) decreasing from 4.35 to 3.70 (Table 3). Although the model with covariates exhibited lower bias (ME = 2.56% *Pf*PR) it also had marginally lower precision (MAE = 4.75% *Pf*PR) compared to the model without covariates. AUC values were similar for both models and indicated acceptable overall fit for endemicity class <5% *Pf*PR (AUC values > 0.70) but less so for endemicty class 5–39% *Pf*PR (AUC values of < 0.70). There were insufficient data points in the validation set to compute AUC values for the endemicity class ≥ 40% *Pf*PR (Table 3).

#### South

In the south the posterior median variance of the spatial process for the model without covariates was 7.14 (95% Bayes CI: 5.00, 8.76) and that of the rate of spatial decay parameter was 4.79 (95% Bayes CI: 2.11, 6.79). For the model with covariates the month of survey, annual average maximum temperature and precipitation all remained significant explanatory factors for *Pf*PR (Table 3). Odds of *P. falciparum *infection were higher in March (OR: 4.06, 95% CI 2.20–7.63) relative to February; and with increasing precipitation (OR: 1.41, 1.07–1.94). Higher minimum temperatures (OR: 0.83, 0.67–0.96) and a survey month of November relative to February (OR 0.48, 0.23–0.96) both reduced the odds of *P. falciparum *infection (Table 3). The inclusion of the covariates improved model fit with DIC of 1,429 compared to 1,454 for the model without covariates. There were no clear differences, however, between the models with and without covariates in the other parameters of model assessment with values of ME (3.65% vs 4.14% *Pf*PR); MAE (5.00% vs 5.06% *Pf*PR) and AUC values (<5% *Pf*PR: 0.91 vs 0.87; 5–39% *Pf*PR: 0.81 vs 0.78; ≥ 40% *Pf*PR: 0.51 vs 0.56). Similar to the multivariate model in the south, most of the spatial residual variation remained unexplained by the covariates.

Overall, the models for the south (with or without covariates) had higher spatial variances and spatial autocorrelation occurred over larger distances compared to the north (Table 3). In addition, models in the south exhibited better model fit with AUC values higher across all endemicity classes probably due to greater availability and better distribution of data in this zone (Figure 2).

### Predicted (posterior median) *Pf*PR maps

**Figure 3:**
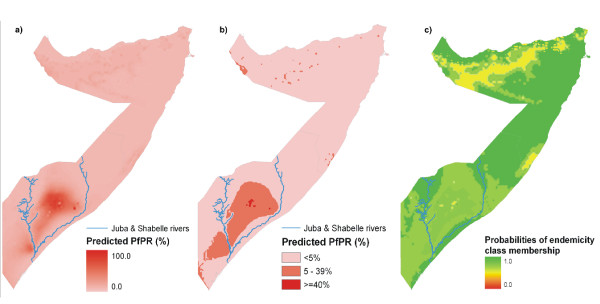
**Maps of north and south of Somalia showing: a) posterior median *Pf*PR; b) endemicity classes based on the posterior probability of *Pf*PR class membership *Pf*PR; c) probability that a prediction location is correctly classified to an endemicity class: <5%; 5–39%; and ≥40% *Pf*PR.** Probabilities < 0.33 are considered no better than chance class assignment. In Somalia probabilities were all ≥ 0.45.

To maintain consistency for comparison, and because the inclusion of the covariates did account for some degree of spatial variation in the *Pf*PR data, the predictions based on the multivariate geostatistical models were retained for both zones (Figures 3a and 3b). Point estimates of *Pf*PR (based on the posterior median) for the prediction locations ranged from 0–9% (median = 0%) in the north. The majority of the area in this zone had predicted a *Pf*PR of <5% with a small number of locations in Puntland and on the south-western border between Somaliland and Ethiopia having *Pf*PR of >5–9% (Figure 3b). In the south, point estimates of *Pf*PR for prediction locations ranged from 0–52% (median 5%), with high *Pf*PR locations occurring in the densely populated farmlands between the Juba and Shabelle rivers (Figure 3b). The lower and upper 95% credible intervals posterior median *Pf*PR are shown in Additional File 3. Interestingly, predicted *Pf*PR was lower along the two rivers, particularly the Shabelle River, compared to the area in between. Overall, predictions of endemicity class membership in the north were associated with lower probabilities compared to those in the south indicating a higher degree of model uncertainty (Figure 3c).

## Discussion

There has been little historical description of the basic epidemiology of malaria transmission in Somalia. In 2002, an application was made to and successfully approved by the GFATM to support the funding of a suite of interventions and strategies managed by a consortium of non-government and governmental agencies across the three main zones of Somalia [51]. This application, similar to other successful applications and RBM policies in neighbouring, higher-intensity transmission countries of Kenya [52], Tanzania [53] and Uganda [54] involved a monitoring & evaluation component to investigate intervention coverage and *P. falciparum *infection rates. In Somalia rapid, sample malaria intervention and parasitological surveys of communities have now become part of a routine component of rolling nutritional surveillance surveys across the country [55]. Consequently, despite being a country without a functioning research capacity and a fragile health system, Somalia is now one of the 87 *P. falciparum *endemic countries worldwide with the largest series of infection prevalence data [56,57].

Simple summaries of the data suggest that large parts of the country, particularly in the north, have very low human infection prevalence (Table 1). These summaries, however, mask spatial heterogeneities in risk that are important for better targeting of interventions and maintaining aggressive surveillance. A Bayesian geostatistical approach to predict *Pf*PR throughout Somalia is used here. In the north, the inclusion of the survey and environmental covariates appeared not to make a significant difference to model fit, while in the south they improved the model fit. Predictions of endemicity class membership made in the north were associated with lower prediction probabilities and generated generally lower AU-ROC values (Table 3 & Figure 3c). This greater prediction uncertainty in the north is due largely to the comparatively fewer empirical data points compared to the south (Figure 2). This disparity was essentially driven by the population distribution: approximately 65% of Somalia's population live in the South and communities in the North are more scattered in isolated settlements [58].

Although the environmental covariates selected for inclusion in the Bayesian geostatistical model were significantly associated with *Pf*PR when examined in the non-spatial multivariate model (Tables 2 &3), none remained significant when spatial correlation was accounted for in the north and only precipitation and temperature remained significant in south. Overall the inclusion of these covariates accounted for a relatively small proportion of spatial variation suggesting that other unmeasured factors might be influencing the spatial distribution of malaria prevalence. These factors might include proximity to artificial breeding sites such as wells, dams, boreholes and seasonal streams and/or the use of interventions to prevent malaria at the household level. It has recently been demonstrated that in southern Somalia, the use of insecticide treated nets (ITN) reduced the prevalence of infection by as much as 54% [30]. Mapping the household or community levels of ITN use at high spatial resolutions is not currently feasible at a national scale. Similarly, the mapping of fluctuating, localized vector breeding sites would require very detailed spatial reconnaissance and infection mapping at finer scales than is currently possible using public domain covariate data at national scales. Furthermore, communities where sample sizes were less than 40, most of which could not be geo-located, were excluded from the analysis and these might have resulted in information loss for some areas of Somalia. Although the difference, in terms of mean parasite prevalence, was minimal between the excluded and included surveys, future analysis should include all data regardless of sample sizes given the Bayesian analytical approach implicitly adjusts for sample size.

Despite the constraints described above, the use of Bayesian geostatistics to model *Pf*PR does provide a valuable method to define sub-national spatial variation in prevalence, and a baseline against which future changes in prevalence can be quantified intervention coverage expands. Under such a scenario the value of the environmental covariates might be expected to wane further, particularly in areas of very low transmission intensity where the environment currently supports homogenously low transmission conditions. The similar levels of performance observed between the univariate and multivariate models for the north of Somalia may be evidence of this view. In addition, the relatively higher coverage of ITN among the communities closest to the two rivers in the south might explain the lower predicted prevalence in their immediate vicinity consistent with the observational data and reported effectiveness of ITN [30].

Population density or a derived categorisation of urbanisation, with known influences on malaria transmission [59,60], would have been a worthy candidate covariate for testing in this study and in determining accurately the population at risk against varying malaria endemicity. However, the reliability of settlement and population data in Somalia is highly questionable. The last national census was undertaken in 1971 and the displacement and migration over the last 20 years of civil unrest has been substantial. Development agencies and non-governmental agencies working in Somalia continue to update a semi-quantitative database of settlement locations and population counts but its fidelity is unknown. The absence of an accurate national census also hampers the linkage of spatial malaria risk to populations-exposed to risk. Notwithstanding the precision and scale of calculating populations at risk, aggregated district-level estimates of population in 2004 across the 120 districts of Somalia have been used and assigned each district the dominant *Pf*PR risk class. From these numbers it can be estimated that approximately 75% of Somalia's estimated 7.4 million people live in areas that support unstable or very low *Pf*PR (0–5%) transmission and less than 0.1% live in areas classified as high, intense transmission (*Pf*PR > 40%). Areas of low *Pf*PR include many communities where infection prevalence was observed as zero (Table 1). In these locations it is assumed that these observations represent a statistical zero (i.e. resulting from a limited sample in areas of very low transmission) rather than implying a true absence of infection risk [56]. This is important to highlight because routine sample surveys in such areas demand considerably larger samples [45,61] or the use of serological markers of parasite exposure [62] to truly exclude the possibility of transmission.

In communities exposed to low *Pf*PR, such as the majority of the population in Somalia, the risk of disease is low and spread across all age-groups. These are fundamentally different epidemiological conditions to areas of high transmission where functional immunity is developed early in life and a higher disease burden is experienced in young children and pregnant women [63-66]. Tailoring the existing intervention recommendations in the Somalia National Malaria Strategy [67] to the spatial transmission patterns shown in Figure 3 will be a challenge to the agencies providing malaria control services nationwide.

## Conclusion

The use of routine, nationwide surveillance of infection prevalence is key to monitoring the changing epidemiology of malaria in all countries scaling up coverage of malaria preventative strategies. Including RDTs in on-going community-based health surveillance is a cost-effective means of assembling this information. The use of geostatistical methods can help focus surveillance efforts and define those areas where uncertainty exists, guiding future sampling [49,68]. Coupled with better estimates of where people live, these should provide the basis for informed estimates of disease burden [63] and how these might change with changing infection-risk exposure. Somalia has a range of political and economic barriers that might limit the success of a strategic, epidemiologically driven malaria control programme. It has been possible to demonstrate, however, that the foci of greatest disease risk are predominantly concentrated in one area in the South and that infection risks are very low in the northern reaches of the country. Moreover, although the density of survey sites and hence the uncertainty of the modelled output varies spatially, also it has been demonstrated that, despite constant civil disturbance, routine survey data can be assembled to inform strategic decision making. Finally, areas where model uncertainties are greatest, predominantly in the north of the country, should be the focus of any future parasitological surveys to improve further the precision of the prevalence maps.

## Abbreviations

AIC: Akaike Information Criterion; AUC-ROC: Area under the curve of the receiver operating characteristic; DIC: Deviance Information Criterion; EVI: Enhanced Vegetation Index; FAO/FSAU: Food Agricultural Organization-Food Security Analysis Unit (FSAU); GFATM: Global Fund to Fight Aids TB and Malaria; ITN: Insecticide treated nets; MAE: Mean absolute error; MAP: The Malaria Atlas Project; MCMC: Markov Chain Monte Carlo; ME: Mean Error; MODIS: Moderate Resolution Imaging Spectroradiometer; *Pf*PR: *Plasmodium falciparum *parasite rate; RDT: Rapid Diagnostics Test; SSA: sub-Saharan Africa; UNICEF: United Nations Children Fund; WHO: World Health Organization.

## Authors' contributions

AMN was responsible for data cleaning, analysis, interpretation and production of the final manuscript and revisions, ACC contributed to the data analysis, interpretation and production of final manuscript, PWG contributed to the data analysis, interpretation and production of final manuscript, GM was responsible for the study design, supervision of data collection, cleaning and contributed to the preparation of the final manuscript, MB provided the necessary interface with community leaders, and the Ministry of Health for approval and was responsible for the data collection, cleaning and contributed to the preparation of the final manuscript, TS assisted in the survey design, supported the field investigations, provided the interface with local ministry of health and helped in the preparation of the manuscript, SIH contributed to overall scientific direction interpretation and preparation of the final manuscript and revisions, RWS was responsible for overall scientific management, analysis, interpretation and preparation of the final manuscript and revisions.

## Supplementary Material

Additional File 1 A method for identifying outliers. Statistical outliers were identified using a spatial filtering algorithm that implemented the following procedure in turn for each datum *p*(*x*_*i*_) at location *x*_*i*_. Click here for file

Additional File 2 The Bayesian model form developed in WinBUGS without covariates. The univariate Bayesian geostatistical models Click here for file

Additional File 3 Maps of north and south of Somalia. Click here for file
