# Supplementary material for: Spatial prediction of Plasmodium falciparum prevalence in Somalia
Source: Malar J. 2008 Aug 21;7:159. doi: 10.1186/1475-2875-7-159 (PMC2531188; doi:10.1186/1475-2875-7-159)
Supplement: Additional File 1 — A method for identifying outliers. Statistical outliers were identified using a spatial filtering algorithm that implemented the following procedure in turn for each datum p(xi) at location xi. [file 1475-2875-7-159-S1.doc]

**Additional File 1: A method for identifying outliers**

Statistical outliers were identified using a spatial filtering algorithm that implemented the following procedure in turn for each datum at location .

1. Around each data location, , a regional neighbourhood was specified defined as the set of data located within five decimal degrees of . This set excluded the datum itself.
2. For each of the unique pairs of data within the regional neighbourhood, the semi-variance was computed along with their separation distance. This set of pair-wise values of and calculated without reference to the datum represented the underlying spatial autocorrelation structure within the regional neighbourhood around location .
3. The next step was to compare the spatial statistical similarity between the datum and it neighbours with the corresponding underlying relationships represented by the regional semi-variance values. Semi-variances were calculated between the datum and its 20 nearest neighbours . Each of these 20 semi-variance values were then compared to regional semi-variance values computed for pairs at similar separation distances, defined as within 0.1 decimal degrees. This required the definition of the subset for each of the semi-variance values .
4. The quantile position of each semi-variance value within the corresponding subset was recorded and the mean of the 20 quantile positions was calculated.

The value provides, for every datum , an index of the extent to which the observed survey datum value differs from its neighbours relative to the extent expected given the distances to those neighbours, as defined by the regional semi-variances. Data for which the value *q_i_* exceeded 0.95 were identified as outliers.
