# Supplementary material for: Spatial prediction of Plasmodium falciparum prevalence in Somalia
Source: Malar J. 2008 Aug 21;7:159. doi: 10.1186/1475-2875-7-159 (PMC2531188; doi:10.1186/1475-2875-7-159)
Supplement: Additional File 2 — The Bayesian model form developed in WinBUGS without covariates. The univariate Bayesian geostatistical models [file 1475-2875-7-159-S2.doc]

**Additional File 2: the Bayesian model form developed in WinBUGS without covariates**

The univariate Bayesian geostatistical models took the form of


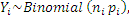


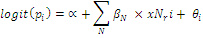


where Y_i_ is the observed number positive at location *i*, n*_i_* is the number tested at location *i*, p*_i_* is predicted prevalence at location *i*, α is the intercept, is a vector of N predictor variables (in the case of this study only survey month was used as covariate), measured a location *i* and *β* are the coefficients. The spatial residual component θ was defined by a powered exponential correlation function:


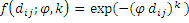


where *d_ij_* are the distances between pairs of points *i* and *j*, *φ* is the rate of decline of spatial correlation with distance and *k* is the degree of spatial smoothing. Non-informative priors were specified for the intercept; normal priors for the coefficients; and uniform prior for *φ.*

For each model a burn in of 1000 iterations was run after which samples statistics were monitored. Convergence occurred after 10,000 iterations for the geostatistical univariate models and 20,000 for the multivariate models for both the south and the north. Convergence was considered to have occurred where Monte Carlo Error/ Standard Deviation was <0.05 for each variable.

Predictions were undertaken on 5 × 5 km grid as a compromise between computational speed and spatial resolution. Prediction was implemented using the *spatial.pred* function in WinBUGS.
