# Supplementary material for: Spatial prediction of Plasmodium falciparum prevalence in Somalia
Source: Malar J. 2008 Aug 21;7:159. doi: 10.1186/1475-2875-7-159 (PMC2531188; doi:10.1186/1475-2875-7-159)

**Additional File 3:** Maps of north and south of Somalia showing: a) the lower 95% credible interval of the posterior median *Pf*PR; b) the upper 95% credible interval of the posterior median *Pf*PR resulting from the multivariate Bayesian geostatistical models. Areas in the North of Somalia where there was scarcity of data had the widest confidence intervals. In the south, the upper limit of the 95% credible interval was approximately 80% and this is related to generally higher prevalence reported in this area.

a) b)


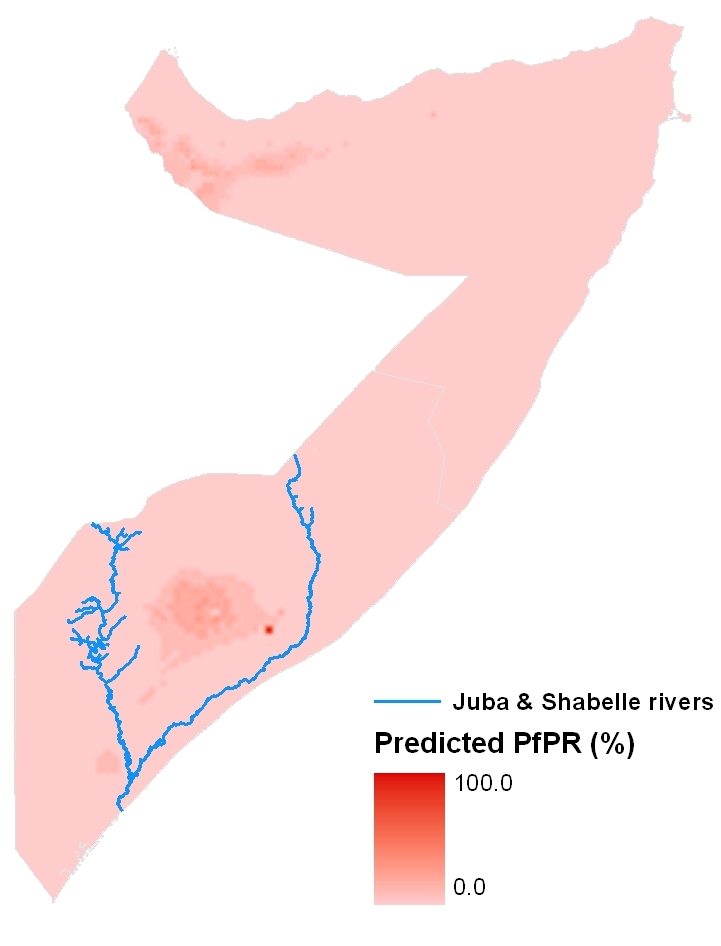

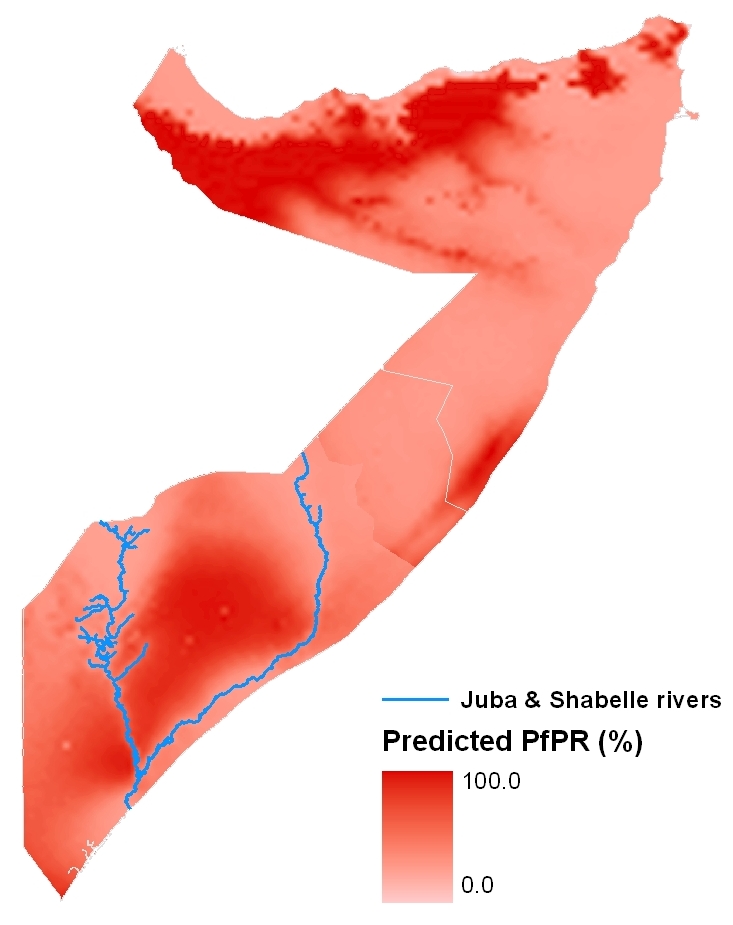

Supplement: Additional File 3 — Maps of north and south of Somalia. [file 1475-2875-7-159-S3.doc]
